# Two-step screening method to identify α-synuclein aggregation inhibitors for Parkinson’s disease

**DOI:** 10.1038/s41598-021-04131-9

**Published:** 2022-01-10

**Authors:** Makoto Hideshima, Yasuyoshi Kimura, César Aguirre, Keita Kakuda, Toshihide Takeuchi, Chi-Jing Choong, Junko Doi, Kei Nabekura, Keiichi Yamaguchi, Kichitaro Nakajima, Kousuke Baba, Seiichi Nagano, Yuji Goto, Yoshitaka Nagai, Hideki Mochizuki, Kensuke Ikenaka

**Affiliations:** 1 Department of Neurology, Osaka University Graduate School of Medicine, 2-2 Yamadaoka, Suita, Osaka 565-0871 Japan; 2 Department of Neurology, Kindai University Faculty of Medicine, 377-2 Ohno-Higashi, Osaka-sayama, Osaka 589-8511 Japan; 3 Global Center for Medical Engineering and Informatics, Osaka University, 2-2 Yamadaoka, Suita, Osaka 565-0871 Japan; 4 Institute for Protein Research, Osaka University, 3-2 Yamadaoka, Suita, Osaka 565-0871 Japan; 5 Department of Neurotherapeutics, Osaka University Graduate School of Medicine, 2-2 Yamadaoka, Suita, Osaka 565-0871 Japan

**Keywords:** High-throughput screening, Parkinson's disease

## Abstract

Parkinson’s disease is a neurodegenerative disease characterized by the formation of neuronal inclusions of α-synuclein in patient brains. As the disease progresses, toxic α-synuclein aggregates transmit throughout the nervous system. No effective disease-modifying therapy has been established, and preventing α-synuclein aggregation is thought to be one of the most promising approaches to ameliorate the disease. In this study, we performed a two-step screening using the thioflavin T assay and a cell-based assay to identify α-synuclein aggregation inhibitors. The first screening, thioflavin T assay, allowed the identification of 30 molecules, among a total of 1262 FDA-approved small compounds, which showed inhibitory effects on α-synuclein fibrilization. In the second screening, a cell-based aggregation assay, seven out of these 30 candidates were found to prevent α-synuclein aggregation without causing substantial toxicity. Of the seven final candidates, tannic acid was the most promising compound. The robustness of our screening method was validated by a primary neuronal cell model and a *Caenorhabditis elegans* model, which demonstrated the effect of tannic acid against α-synuclein aggregation. In conclusion, our two-step screening system is a powerful method for the identification of α-synuclein aggregation inhibitors*,* and tannic acid is a promising candidate as a disease-modifying drug for Parkinson’s disease.

## Introduction

Parkinson’s disease (PD) is the second most common neurodegenerative disease after Alzheimer’s disease (AD), and the number of patients is increasing with the aging of the global population^1^. PD is clinically characterized by progressive motor and non-motor symptoms. Although the currently available dopamine replacement therapies can ameliorate some of the symptoms, no curative therapy is available at present, and symptoms become disabling as the disease progresses. Therefore, disease-modifying therapies that slow or halt disease progression are urgently required.

The pathological hallmarks of PD include the appearance of neuronal inclusions called Lewy bodies, which are mainly composed of aberrant α-synuclein (αSyn) aggregates, and progressive neuronal loss mainly involving dopaminergic neurons in the substantia nigra^2^. Cumulative evidence has indicated that pathological αSyn and αSyn aggregates play a central role in the pathogenesis of PD, and its cell-to-cell propagation is associated with disease progression^3^. Therefore, the suppression of αSyn aggregation and/or the depolymerization of αSyn aggregates have been considered to be promising therapies for PD. Potential drugs that target αSyn aggregation act via the following strategies: suppression of αSyn expression^4,5^, modulation of the protein degradation machinery^6^, inhibition of the aggregation of αSyn^7,8^, and depolymerization of αSyn fibrils^9^. Among them, inhibition of the aggregation of αSyn is the most straightforward strategy that targets the pathological process underlying PD^10,11^. High throughput screening (HTS) of 1000 pharmacophores with the second-harmonic generation method identified BIOD303 as a modulator of the conformation of monomeric αSyn which reduced αSyn aggregation^11^. Recently, HTS by the thioflavin T (ThT) assay identified SynuClean-D^12^ and ZPD-2^13^ as αSyn aggregation inhibitors from 14,400 compounds of Maybridge HitFinder Collection^14^. Although these reports have shown that HTS for αSyn aggregation inhibitors is a feasible strategy to identify drug candidates for PD, the cell-free screening methods employed in these studies could have not properly evaluated the influence of the cellular milieu on the action of the tested compounds. Moreover, ThT assay only monitors the fibrillation process of αSyn monomer, whereas the desired candidate compounds for patients is to inhibit the propagation of existing fibrils. To overcome these limitations, we propose a two-step screening method consisting of the conventional HTS ThT assay followed by a cell-based assay that allows the evaluation of seeding capability of the fibrils.

In this study, we evaluated 1262 FDA-approved compounds to enable future drug repositioning of the candidates. We took advantage of the high-throughput nature of the ThT assay, together with a cell-based system, which can evaluate both the efficacy and safety of the candidates. We identified seven compounds, and the robustness of our strategy was validated by the analysis of tannic acid (TA), which was the most effective molecule identified in our screening.

## Results

### ThT fluorescence assay for the screening of αSyn fibrillation inhibitors

Pujols et al. reported that the ThT fluorescence assay was the robust method to evaluate the kinetics of fibrillation of amyloid proteins including αSyn, and was useful for screening of αSyn fibrillation inhibitors^14^. In this study, we used this method as our first step of screening. Amyloid formation of αSyn was observed as a sigmoidal increase in ThT fluorescence (Fig. S1A), as previously described^14^. We then corroborated that the presence of ThT did not affect the kinetics of fibril formation (Fig. S1B) or the fibril morphology, visualized by transmission electron microscopy (TEM) (Fig. S1C). Also, the increase in ThT fluorescence was suppressed by the addition of Congo Red, which is known to have an inhibitory effect against αSyn fibrillation (Fig. S1D). For the quantitative evaluation of fibrillation, we determined the lag time until the ThT fluorescence intensity exceeded 1000 A.U., and the maximum ThT intensity. The lag time was counted as 2505 min if the fluorescence intensity did not exceed 1000 A.U., which was the time we finished the experiments. Congo Red significantly delayed the lag time (Fig. S1E) and inhibited the formation of amyloid fibrils (Fig. S1F).

**Figure 1:**
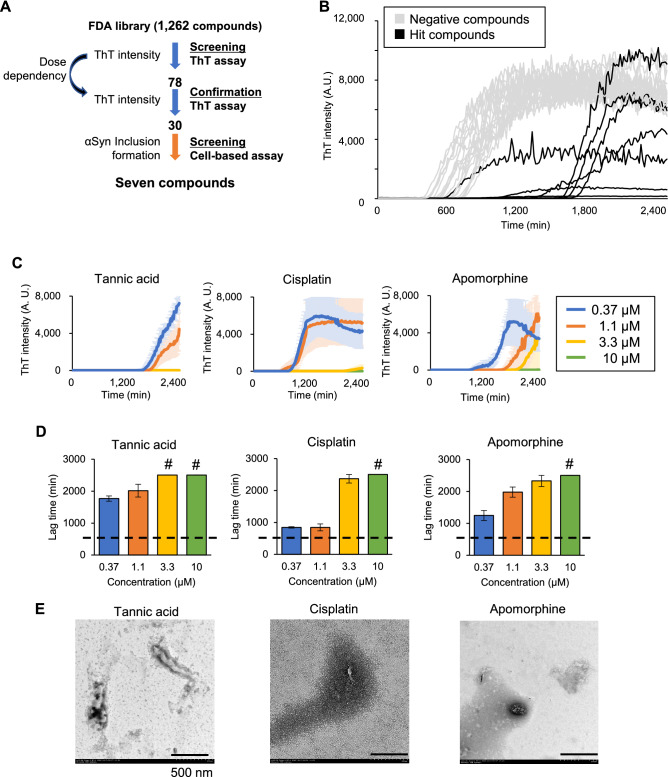
ThT fluorescence assay used for the screening of αSyn fibrillation inhibitors. (**A**) Schematic illustration of the two-step screening. (**B**) ThT kinetics of the wells with representative positive hits (black lines) and negative compounds (gray lines). (**C**) ThT kinetics of the top 3 hit compounds at different concentrations. Data are shown as the mean ± SEM of three independent wells (n = 3). Concentrations of the drugs are as indicated in the right box. (**D**) Representative lag times of the top 3 hit compounds at different concentrations. Dashed line represents the average lag times in the absence of any compound. Sharp marks (#) indicate that the reaction was stopped since it reached to the maximum duration (2505 min). Data are shown as the mean ± SEM of three independent wells (n = 3). (**E**) TEM visualization of fibrillation reaction products treated with the indicated compounds. Bar scale: 500 nm.

We screened 1262 FDA-approved compounds by the ThT assay as a first step of the screening for αSyn aggregation inhibitors, then, after confirming the dose-dependency of the positive compounds, we analyzed their effects using the cell-based assay (Fig. 1A). A single concentration (10 μM) for all the compound was tested in duplicate in the ThT assay. We established the lag time and the value of maximum ThT intensity within a same plate as the criteria to evaluate the efficacy of the tested compounds to suppress fibrillation. A compound was considered a hit when any of both parameters was found within the top ten percentile in the duplicate assay. Representative ThT kinetics of the wells with positive and negative compounds are shown in Fig. 1B. The 78 compounds that were positive in the first screening at the concentration of 10 μM were analyzed in the next screening using four different compound concentrations (10, 3.3, 1, and 0.37 μM), tested in triplicate. We ranked the compounds according to their lag time at 10 μM. If the lag time values at 10 μM were not different between compounds, we used the lag time at 3.3 μM, or lower concentrations until a difference was observed. The top 30 hits are listed in Table 1. Representative ThT kinetics and the lag times of the top 3 compounds at different concentrations are shown in Fig. 1C,D. The inhibition of fibril formation was confirmed by TEM (Fig. 1E).

**Table 1:** Top 30 hit compounds identified in the ThT screening assay for αSyn fibrillation inhibitors.

	Compound	Mean lag time (min) n = 3			
		0.37 μM	1.1 μM	3.3 μM	10 μM
1	Tannic acid	1770	2015	2505	2505
2	Cisplatin	845	845	2370	2505
3	Apomorphine hydrochloride	1245	1980	2330	2505
4	Norepinephrine	1125	1530	2040	2505
5	Diflunisal	1730	1850	1965	2505
6	Rabeprazole sodium	985	1665	2015	2500
7	Althiazide	1120	1555	1780	2200
8	Dopamine hydrochloride	1065	1295	1620	2085
9	Hymechrome	1800	1705	1820	2070
10	Pantoprazole	950	1215	1785	2070
11	Tamoxifen citrate	1255	1305	1515	2070
12	Donepezil hydrochloride	1345	1340	1560	2030
13	Hyoscyamine	1405	1450	2195	2010
14	Glucosamine hydrochloride	1190	1760	2095	1925
15	Methyldopa	1440	1415	1240	1925
16	Chlorophyllide Cu complex Na salt	820	930	1000	1920
17	Isotretinoin	1495	1485	2050	1885
18	Pirenperone	1355	1450	1865	1845
19	Entacapone	1385	1695	1830	1800
20	Nisoldipine	1285	1450	1490	1795
21	Chloroxine	1345	1445	1875	1785
22	Paroxetine hydrochloride	1330	1385	1620	1705
23	Risedronate sodium	1260	1520	1640	1700
24	Nateglinide	955	1370	1360	1630
25	Diethylcarbamazine citrate	1060	1350	1470	1625
26	Deferoxamine mesylate	1260	1335	1255	1580
27	Fenoldopam mesylate	1120	1180	1390	1400
28	Oxidopamine hydrochloride	1045	1055	1125	1365
29	Oxyquinoline sulfate	575	725	815	1060
30	Neomycin sulfate	670	815	810	980

### Cell-based assay for the screening of αSyn aggregation inhibitors

**Figure 2:**
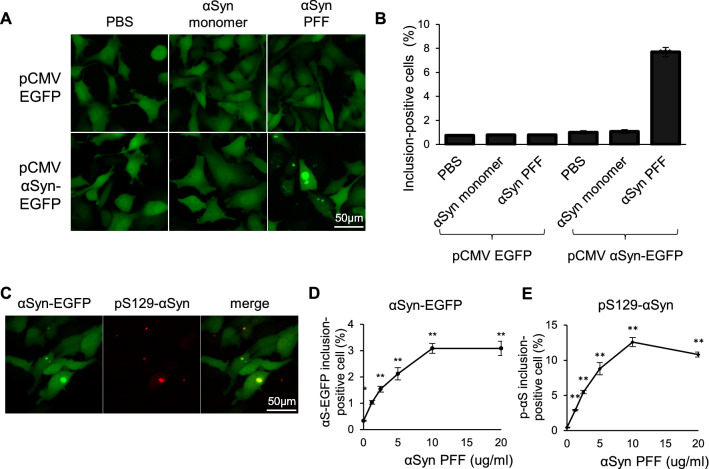
Establishment of a cell-based assay to evaluate αSyn aggregation. (**A**) Representative images of αSyn-EGFP aggregation in HeLa cells. HeLa cells were transfected with the indicated plasmids, and then treated with PBS, αSyn monomers, or PFF. (**B**) Quantification of the percentage of cells containing obvious αSyn-EGFP inclusion bodies in (**A**). Data are shown as the mean ± SEM of twelve independent wells (n = 12; **P < 0.01; two-way ANOVA with the Tukey test). (**C**) Representative immunocytochemistry images of PFF-treated HeLa cells. Cells were transfected with pCMV αSyn-EGFP, and then treated with αSyn PFF. Phosphorylated αSyn (pS129 αS) was stained using a specific antibody. (**D**,**E**) Quantification of the percentage of cells containing obvious αSyn-EGFP (**D**) and p129-αSyn (**E**) inclusion bodies. Data are shown as the mean ± SEM of four independent wells (n = 4; *P < 0.05; **P < 0.01; one-way ANOVA with the Dunnett test compared with the no αSyn PFF control).

Exogenous αSyn preformed fibril (PFF) can seed the formation of Lewy body-like intracellular inclusions in cultured cells^15^. Based on this report, we developed a 96-well plate cell-based assay coupled with an automatic quantification system to further analyze the effects of the hit compounds identified by the ThT screening. To create cellular models of αSyn aggregation, we transiently overexpressed αSyn conjugated with enhanced green fluorescent protein (αSyn-EGFP) in HeLa cells, and treated the cells with PFF of αSyn. We confirmed the formation of large intracellular inclusions only when cells were treated with both αSyn-EGFP overexpression and αSyn PFF (Fig. 2A). The percentage of cells containing obvious EGFP inclusion bodies was significantly higher in the αSyn-EGFP overexpressing cells treated with αSyn PFF than in the other groups (Fig. 2B). The number of nuclei (Fig. S2A) and the percentage of EGFP-positive cells (Fig. S2B) were not significantly altered by PFF treatment. To investigate the biological relevance of the EGFP inclusions, we stained for αSyn phosphorylated on Ser129 (pS129 αS), which is considered to be the pathological form of αSyn. We found that αSyn-EGFP inclusions colocalized with pS129 αS (Fig. 2C). The percentage of cells containing EGFP inclusions and pS129 αS increased in a concentration-dependent manner up to 10 μg/mL of αSyn PFF (Fig. 2D,E). Taken together, our cell-based assay evaluating αSyn-EGFP inclusions was confirmed to detect changes in the amount of aggregation of pathological αSyn, and it is hence useful for drug screening.

**Figure 3:**
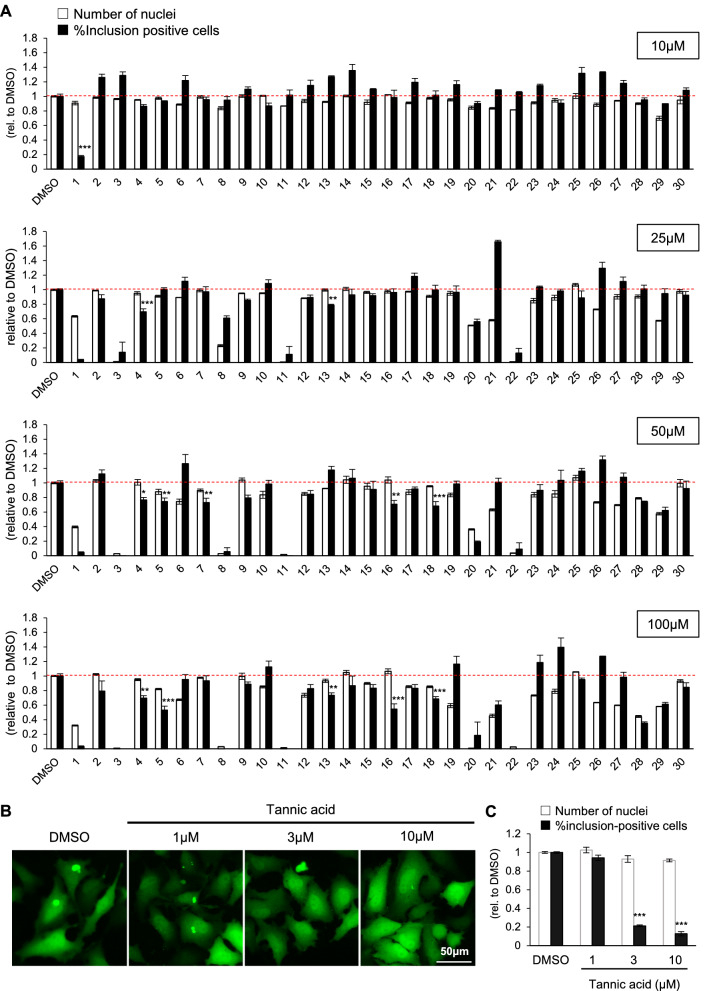
Cell-based screening of αSyn aggregation inhibitors. (**A**) Quantification of the number of nuclei (white bars) and the percentage of cells containing obvious αSyn-EGFP inclusion bodies (black bars), standardized by those of cells treated with DMSO. Concentration of the drugs are indicated at the right. The numbers under the graph correspond to the numbers in Table 1. Data are shown as the mean ± SEM of three independent wells (n = 3; *P < 0.05; one-way ANOVA with the Dunnett test compared with the DMSO control). (**B**) Representative images of tannic acid-treated HeLa cells. Cells were transfected with pCMV αSyn-EGFP, followed by treatment with αSyn PFF and the indicated concentrations of tannic acid. (**C**) Quantification of the number of nuclei (white bars) and the percentage of cells containing obvious αSyn-EGFP inclusion bodies (black bars) in B standardized by those of cells treated with DMSO. Data are shown as the mean ± SEM of three independent wells (n = 3; *P < 0.001; one-way ANOVA with the Dunnett test compared with the no tannic acid control).

As in previous reports, we used the αSyn aggregation inhibitor rifampicin as a positive control in our assay^16^. Our cell-based assay demonstrated that rifampicin reduces the percentage of cells containing EGFP inclusions in a dose-dependent manner (Fig. S3, black bars). On the other hand, the dose-dependent decrease in the number of nuclei suggested that rifampicin is cytotoxic (Fig. S3A, white bars). Considering that the optimal concentration may be different among drugs, we tested four concentrations (10, 25, 50, and 100 μM) for each compound (Fig. 3A). A compound was defined as a hit by its efficacy (a significant reduction in αSyn-EGFP inclusions), in combination with its safety (< 20% cell loss). As a result, the following seven drugs were finally selected as hit compounds: TA, norepinephrine, diflunisal, althiazide, hyoscyamine, chlorophyllide Cu complex Na salt, and pirenperone (Table 2). We confirmed that at an optimal concentration of these compounds, there was no significant cell toxicities (Fig. S3B). Among them, TA showed the most prominent reduction in the number of αSyn-EGFP inclusions. TA also inhibited αSyn inclusions in a dose-dependent manner (Fig. 3B,C). Additionally, in order to exclude the possibility that TA disassembles mature fibrils in the extracellular space, we incubated PFF with TA for 24 h and then evaluated the morphology by TEM, confirming that the fibrils were stable in the presence of TA (Fig. S3C).

**Table 2:** Seven final αSyn aggregation inhibitor candidates identified by the two-step screening.

	Compound	%Inhibition	%Viability	Conc. (μM)
1	Tannic acid	83	91	10
4	Norepinephrine	30	95	100
5	Diflunisal	47	82	100
7	Althiazide	27	90	50
13	Hyoscyamine	26	93	100
16	Chlorophyllide Cu complex Na salt	46	106	100
18	Pirenperone	32	85	100

### TA inhibits aSyn aggregation in primary cultured neuronal cells and a *C. elegans* PD model

**Figure 4:**
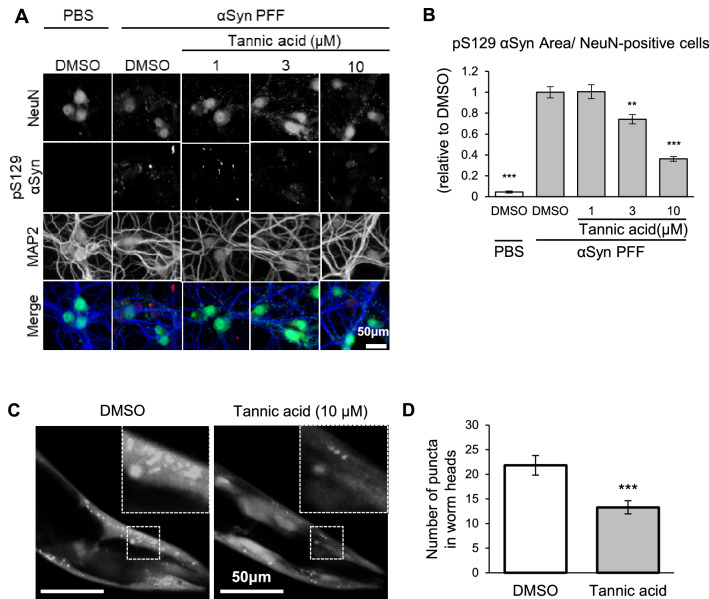
Tannic acid inhibits the formation of αSyn aggregates in mouse primary cultured neurons and PD model *C. elegans*. (**A**) Representative immunocytochemistry images of primary cultured neuronal cells treated with αSyn PFF and tannic acid. Primary neurons were treated with the indicated combinations at DIV 7, and were stained at DIV 14. (**B**) Quantification of pS129 αSyn in PFF- and tannic acid-treated neurons. Relative pS129 αSyn area per number of NeuN-positive neuronal cells was standardized by that of cells treated with αSyn PFF and DMSO. Data are shown as the mean ± SEM of three independent wells (n = 3; **P < 0.01; ***P < 0.001; one-way ANOVA with the Dunnett compared with the αSyn PFF without tannic acid control) (**C**) Representative images of the heads of PD model *C. elegans* treated with DMSO or tannic acid. (**D**) Quantification of the number of obvious puncta in *C. elegans* treated with tannic acid or the DMSO control*.* Data are shown as the mean ± SEM of twenty-eight worms (n = 28; ***P < 0.001; Student *t*-test).

Next, to investigate the effects of TA on neuronal cells, which are the cells mainly affected by αSyn in Parkinson’s disease, we used primary cultured neurons from embryonic mouse cortex. The endogenous α-synuclein in mouse neurons is subject to aggregation and phosphorylation when exogeneous α-synuclein PFF is administered^17^. Treatment with αSyn PFF and TA started on days in vitro (DIV) 7 and the cells were fixed after 7 days (DIV 14). The level of pS129 αS per neuron significantly decreased with TA treatment in a dose-dependent manner, indicating that TA may prevent the PFF-induced aggregation of endogenous αSyn in mouse neurons (Fig. 4A,B). Then, to investigate the effects of TA in vivo, we used the NL5901 strain of *C. elegans*, which has been established as a model for PD. In NL5901 worms, expressing αSyn-YFP in muscle cells, αSyn forms aggregates with aging^18^. Age-synchronized 3-day-old young adult worms were transferred to a plate containing 10 μM TA, and the number of aggregates in 6-day-old wormswere evaluated. The number of aggregates was suppressed by about 30% upon treatment with TA (Fig. 4C,D).

## Discussion

In this study, we used a two-step screening method to find candidate αSyn aggregation inhibitors. Taking advantage of the high-throughput ability of our method, we first evaluated 1262 FDA-approved compounds by the ThT assay and selected 30 hit compounds. To evaluate the efficacy and safety of these compounds in the cell-based assay, we developed a 96 well-based automatic quantification system to detect αSyn-EGFP inclusions. This system facilitated the analysis of several distinct concentrations of the compounds at one time, resulting in an increased chance to identify hit compounds. Seven out of 30 candidates (23.3%) were identified as potential αSyn aggregation inhibitors by this second step. A high hit-ratio of the second screening indicated the usefulness of the first ThT assay. Of note, the other 23 compounds were unable to inhibit αSyn aggregation in the cellular milieu owing to a weaker efficacy than expected (15/23, 65%) and/or cell toxicity (15/23, 65%). The discrepancy in compound efficacy between the ThT assay and the cell-based model may be mainly attributed to the complex environment within the cellular milieu. Compared with the simplicity of the in vitro ThT assay, the dynamics of αSyn in the living cell will be affected by various other molecules and protein quality control systems in vivo. The membrane permeability, intracellular modification, and metabolism of the compounds may also affect the results. Therefore, substances that exert their effects in the cell-based assay can be considered as promising candidates. Taken together, our two-step screening system may be a feasible strategy to identify drug candidates for inhibitors of pathological αSyn aggregation.

Our screening of 1262 FDA-approved compounds identified TA as the most effective candidate to inhibit αSyn aggregation. TA showed a reduction of 80% or more in αSyn inclusion-positive cells without any significant cytotoxicity. TA is a polyphenol compound, and previous studies have demonstrated that some polyphenols, including TA, suppress the aggregation of αSyn^19–21^. Therefore, the identification of TA from our screening contributes to validate the robustness of our screening system.

In this study, the efficacy of TA as an αSyn aggregation inhibitor was further validated in neuronal cells and a *C. elegans* model of PD. To the best of our knowledge, this is the first report showing the effects of TA on the inhibition of αSyn aggregation in a neuronal model and an animal model. As TA also suppresses Aβ aggregation, which is a hallmark of AD, this compound might have the potential of broad application to many neurodegenerative diseases. Interestingly, it has been reported that the oral administration of TA to an AD mouse model reduced Aβ deposits in the brain, although TA itself is not expected to cross the blood–brain barrier (BBB). This suggests that some metabolites of TA might remain active and pass across the BBB. Moreover, we also expect that the inhibition of αSyn aggregation in peripheral organs might exert beneficial effects on PD pathology. Cumulative reports have suggested the progression of PD pathology through the transmission of αSyn aggregates from the gut and olfactory bulb to the brain. From this point of view, the suppression of αSyn aggregation in the peripheral organs, such as the gut by TA may ameliorate brain pathology. Further detailed investigation of the effects of TA on PD and other neurodegenerative disorders is required in the future.

Other than TA and other polyphenol compounds, we found diflunisal, an FDA-approved drug for transthyretin (TTR) familial amyloid polyneuropathy^22^. Diflunisal inhibits TTR aggregation by stabilizing the tetrameric conformation of TTR^23,24^. Interestingly, αSyn is more stable and resistant to aggregation in its tetrameric form^25^. Further studies will be required to confirm the action of diflunisal on αSyn aggregation.

The limitations of our study include the following points: first, the two assays performed were based on artificial conditions, while the hit compounds will require a further validation under more physiological conditions. In this regard, we have already investigated the effects of TA in neuronal cells without performing transfection, and in nematodes without adding PFF. Second, we did not test a sufficient range of concentrations for all the compounds in the ThT assay, just a single concentration of 10 µM, which could result in false-negative results. In addition, the cell-based assay may also have produced false negative results since some compounds were not effective but still not toxic at the maximum concentration of 100 μM.

In summary, our two-step screening of 1262 FDA-approved compounds identified seven candidate drugs as αSyn aggregation inhibitors. In particular, TA appeared to be the most promising of the candidates, and further studies towards its clinical application are expected in the future.

## Materials and methods

### Plasmids

The plasmid containing the human αSyn was originally created in a previous study by amplification from cDNA of human brain (Cap site cDNA dT: Nippon gene) by PCR^26^. The MultiSite Gateway^®^ donor vectors (Invitrogen, MA, USA) were used to clone human wild type (WT) αSyn to generate the entry clones pENTR-L1-ACC-αSyn and -R5. Entry clones were then recombined with pcDNA™-DEST40 (Invitrogen) and pENTR-L5-GGS6-EGFP-L2^27^ (a gift from Professor H. Kuroyanagi) to create the final expression clone αSyn-GGS6-EGFP.

### Preparation of αSyn

Human WT αSyn was purified from *Escherichia coli* (*E. coli*) as described previously^28^. Briefly, a plasmid containing WT human αSyn was expressed in *E. coli* BL21 (DE3) (Novagen, Merck, San Diego, CA, USA). The cells were suspended in purification buffer, disrupted by sonication, and centrifuged. Streptomycin sulfate (final 2.5% [w/w]) was added to the supernatant and centrifuged. The supernatant was then heated at 90 °C in a water bath and centrifuged. The supernatant was precipitated by solid ammonium sulfate to 70% saturation, centrifuged, dialyzed overnight, and applied onto a Resource-Q column (GE Healthcare, Little Chalfont, UK) with 50 mM Tris–HCl buffer (pH 7.5) containing 0.1 mM dithiothreitol and 0.1 mM phenylmethylsulfonyl fluoride as the running buffer, and eluted with a linear gradient of 0 to 1 M NaCl. αSyn-enriched fractions (as determined by sodium dodecyl sulfate–polyacrylamide gel electrophoresis/Coomassie blue staining) were pooled and further purified by size exclusion chromatography using a Superdex 200 10/300 GL column (GE Healthcare) equilibrated with 50 mM Tris–HCl (pH 7.5) and 150 mM NaCl. The purified fractions were combined and dialyzed against deionized water at 4 °C overnight. Sample solutions were flash-frozen in liquid nitrogen and lyophilized.

### In vitro fibrillation of αSyn

The in vitro fibrillation assay was performed as reported previously^14^, with some modifications. Briefly, lyophilized αSyn was dissolved in a buffer containing 250 mM NaCl, 50 mM Tris–HCl (pH 7.4), 10 μM ThT, filtered through a 0.22 µm membrane to remove αSyn aggregates, and adjusted to a final concentration of 500 μg/mL. For the evaluation of ThT concentration dependency (Fig. S1B), the concentration of ThT ranged between 1 and 20 µM. The fibrillation reaction was carried out in a 96-well sealed plate (Costar Assay Plate, Corning, USA). Each well contained 100 μL of reaction mixture with or without compounds at 10 µM. The reaction 96-well microplate was subjected to cyclic agitation with a 3 min orbital shaking period at 2000 rpm, followed by a 12 min quiescent period, at 37 °C. Amyloid formation was monitored by ThT intensity fluorescence (excitation at 450 nm and emission at 485 nm) every 15 min. Fluorescence measurement and shaking procedures were performed using a MTP-900 microplate reader (Corona Electric Co., Tokyo, Japan). All the samples were measured in duplicate, and the formation of fibrillar aggregates was characterized by the lag time, defined as the time required to reach a fluorescence value of 1000 A.U., and the maximum ThT intensity, the highest intensity value in the measuring period, as the parameters in αSyn fibrillation.

### TEM analysis

Fibrils were adsorbed onto 400-mesh grids and negatively stained with 1% phosphotungstic acid, and their structures were observed using an H-7650 TEM (Hitachi High Technologies Corporation, Tokyo, Japan).

### Cell culture

HeLa cells were purchased from KAC (KAC, Kyoto, Japan). Cells were cultured in Dulbecco’s Modified Eagle’s Medium (Sigma-Aldrich, MO, USA, D5796) supplemented with 10% fetal bovine serum (FBS) at 37 °C in a 95% air, 5% CO_2_ humidified incubator. Cells were routinely subcultured when confluent. The maximum passage of the cell line was 20 times.

### Creation of a cell-culture model of αSyn aggregation

HeLa cells were transfected with the αSyn-EGFP plasmid using FuGENE HD Transfection Reagent (Promega, WI, USA) following the manufacturer’s instructions, to overexpress αSyn. After 24 h, the culture medium was replaced with culture medium containing αSyn PFF with or without the drug compounds. After 24 h of incubation, cells were fixed with 4% paraformaldehyde (Wako, Japan) for 30 min at room temperature, and then the cells were immunostained for pS129 αS and microscopically analyzed.

### Immunostaining

Fixed HeLa cells were washed in phosphate-buffered saline (PBS), then incubated in PBS with 10% Block Ace (Yukizirushi, Tokyo, Japan) for 1 h and subsequently with primary anti-phosphorylated αSyn (pS129 αS) (1:1000; Abcam, Cambridge, UK) for 4 °C overnight. After several washes in PBS, the cells were incubated with Cy3-conjugated anti-rabbit antibody (Jackson ImmunoResearch, PA, USA) for 1 h at room temperature. After several washes in PBS, the cells were counterstained with Hoechst 34580 (Invitrogen).

### Image acquisition and analysis

Images of the treated cells were obtained using IN Cell Analyzer (GE Healthcare). Thirty-six images of a culture well were taken at 20× magnification. Automated quantification of the number of nuclei, cells expressing EGFP, and cells expressing αSyn-EGFP, or with pS129 αS inclusions were performed using the software IN Cell Developer Toolbox (GE Healthcare).

### Cell toxicity assay

HeLa WT cells were incubated with the optimal concentration of compounds as listed in Table 2 for 24 h and the number of nuclei were counted as described above.

### Primary neuronal culture model of αSyn aggregation and TA treatment

Pregnant C57BL/6J mice, (RRID:IMSR_JAX:000664) were obtained from Charles River Laboratories Japan (Yokohama, Japan). Primary cortical neurons were prepared from E15.5 pups and cultured in MACS Neuro Medium (Miltenyi Biotec, Bergisch Gladbach, Germany) supplemented with MACS NeuroBrew-21 (Miltenyi Biotec), 0.5 mM l-glutamine, penicillin, and streptomycin (all from Invitrogen) on tissue culture plates coated with poly-l-ornithine (Fujifilm, Tokyo, Japan) at a density of 5 × 10^4^ cells/cm^2^. At 3 days in vitro (DIV), a half volume of fresh media containing 2′-deoxy-5-fluorouridine (final concentration, 3.3 µM) (Tokyo Chemical Industry, Tokyo, Japan) was added to inhibit glial proliferation. At 7 DIV, half of the media was changed, and αSyn PFF (final concentration, 10 µg/mL) with or without TA was added. αSyn PFF were incubated for 7 days and then analyzed by immunocytochemistry.

### *C. elegans* experiments

Standard methods were used to culture *C. elegans* on nematode growth medium (NGM) agar seeded with OP50 *E. coli*, as previously described^29,30^. The worms were maintained at 20 °C unless otherwise indicated.

N2 WT (Bristol) and NL5901^18^ were obtained from the *Caenorhabditis elegans* Genetic Center.

### Quantification of αSyn aggregates formed in *C. elegans*

The quantification of aggregates was performed as previously described^12,18^ with brief modifications. Briefly, NL5901 (*Punc-54:α-SYN:YFP*) worms were age-synchronized and left overnight to hatch. Synchronized nematodes were cultured and grown on NGM plates seeded with *E. coli* OP50 until they reached the young adult stage (day 3). Afterwards, animals were transferred onto 5-Fluoro-2′-deoxyuridine-containing plates with or without TA (10 μM). αSyn aggregates were counted for each animal 3 days after treatment. For each independent experiment, 28 worms for each treatment were examined under a Zeiss LSM 700 microscope. Aggregates were defined as discrete bright structures with boundaries distinguishable from the surrounding fluorescence. Measurements of the aggregates were performed visually on all the aggregates observed in the head region of the worms. The experiments were performed blinded. The experimenter who performed the quantification was unaware of the worm’s group.

### Statistical analysis

All data were analyzed using JMP software. For the statistical analysis of 2 groups, the paired or unpaired t-test was used as described in the figure legends. In all experiments, data were expressed as the mean ± SD and a p value of < 0.05 was considered to indicate a statistically significant difference between 2 groups. One-way analysis of the variance (ANOVA) with the Dunnett post-hoc test for more than three groups.

### Statement

All animal experiments were conducted with approval from the Animal Care and Use Committee and the Committee for Safe Handling of Living Modified Organisms of the Graduate School of Medicine, Osaka University. This study was carried out in compliance with the ARRIVE guidelines. All methods were carried out in accordance with relevant guidelines and regulations.

## Supplementary Information

Supplementary Figures.

## Abbreviations

αSyn: α-Synuclein
DMSO: Dimethyl sulfoxide
FDA: Food and drug administration
PD: Parkinson’s disease
PFF: Preformed fibril
RRID: Research Resource Identifier
TA: Tannic acid
