# Supplementary material for: Two-step screening method to identify α-synuclein aggregation inhibitors for Parkinson’s disease
Source: Sci Rep. 2022 Jan 10;12:351. doi: 10.1038/s41598-021-04131-9 (PMC8748996; doi:10.1038/s41598-021-04131-9)

Title: Two-step screening to identify α-synuclein aggregation inhibitors for Parkinson's disease.

Author names:

Makoto Hideshima[1], Yasuyoshi Kimura[1], César Aguirre[1], Keita Kakuda[1], Toshihide Takeuchi[2,5], , Chi-Jing Choong[1], Junko Doi[1], Kei Nabekura[1], Keiichi Yamaguchi[3,4], Kichitaro Nakajima[3], Kousuke Baba[1], Seiichi Nagano[1,5], Yuji Goto[3,4], Yoshitaka Nagai[2,5], Hideki Mochizuki[1], and Kensuke Ikenaka[1]

Affiliation:

[1]Department of Neurology, Osaka University Graduate School of Medicine, 2-2 Yamadaoka, Suita, Osaka 565-0871, Japan

[2]Department of Neurology, Kindai University Faculty of Medicine, 377-2 Ohno-Higashi, Osaka-sayama, Osaka 589-8511, Japan

[3]Global Center for Medical Engineering and Informatics, Osaka University, 2-2 Yamadaoka Suita, Osaka, 565-0871, Japan

[4]Institute for Protein Research, Osaka University, 3-2 Yamadaoka, Suita, Osaka 565-0871, Japan

[5]Department of Neurotherapeutics, Osaka University Graduate School of Medicine, 2-2 Yamadaoka, Suita, Osaka 565-0871, Japan

***Corresponding author:**

Kensuke Ikenaka

Tel: +81-06-6879 3571; Fax: +81-06-6879 3579.

E-mail address: ikenaka@neurol.med.osaka-u.ac.jp; Postal address: Department of Neurology, Yamadaoka 2-2, Suita, Osaka 565-0871, Japan.

and

Hideki Mochizuki

Tel: +81-06-6879 3571; Fax: +81-06-6879 3579.

E-mail address: hmochizuki@neurol.med.osaka-u.ac.jp; Postal address: Department of Neurology, Yamadaoka 2-2, Suita, Osaka 565-0871, Japan.

Supplementary Figure legends

Supplementary Figure 1. Validation of the thioflavin T fluorescence assay for the evaluation of α-synuclein aggregation

(A) ThT-monitored aSyn fibril formation in the presence of 10 μM ThT. (B) Kinetic analysis of the formation of fibrils in the presence of 1-20 μM ThT performed employing the parameter $t_{1/2}$. (C) TEM visualization of amyloid fibrils created in the presence of 1 and 10 μM ThT. Scale bar, 200 nm. (D) Thioflavin T (ThT) kinetics of the samples in the absence (black line) or presence (gray line) of Congo Red. Data are shown as the mean ± SEM (DMSO, n = 80 wells; Congo Red, n = 16 wells). (E) Quantification of the lag time from the data in (A). Data are shown as the mean ± SEM (DMSO, n = 80 wells; Congo Red, n = 16 wells; ***$P < 0.001$; Student *t*-test). (F) Transmission electron microscopy (TEM) images of α-synuclein (αSyn) samples after incubation in the absence (left) or presence (right) of Congo Red. Scale bar, 200 nm

Supplementary Figure 2. Evaluation of cytotoxicity and transfection efficiency of our cell-based assay

Quantification of the number of nuclei (A) and the percentage of cells expressing EGFP (B) after plasmid transfection and αSyn treatment in our cell-based assay. Data are shown as the mean ± SEM. NS indicates no significance (n = 12; two-way ANOVA with the Tukey test). Please refer to Figure 2.

Supplementary Figure 3. Validation of our cell-based assay for the evaluation of α-synuclein aggregation

(**A**) Rifampicin inhibits the formation of αSyn-EGFP inclusions in our cell-based assay. Number of nuclei (white bars) and the percentage of cells containing obvious αSyn-EGFP inclusion bodies (black bars) in cells treated with rifampicin standardized by those of cells treated with DMSO as a control, in our cell-based assay. Data are shown as the mean ± SEM (n = 4; *$P < 0.05$; **$P < 0.01$; ***$P < 0.001$; one-way ANOVA with the Dunnett test compared with the control). (B) Hit compounds at an optimal concentration cause no significant cell toxicities. Quantification of the number of nuclei after drug exposure without αSyn treatment in our cell-based assay. Data are shown as the mean ± SEM. NS indicates no significance (n = 4; one-way ANOVA with the Dunnett test compared with the control).(C) TEM visualization of fibrils treated for 24 hours with and without TA. Scale bar, 200 nm.

Supplementary Figure 1

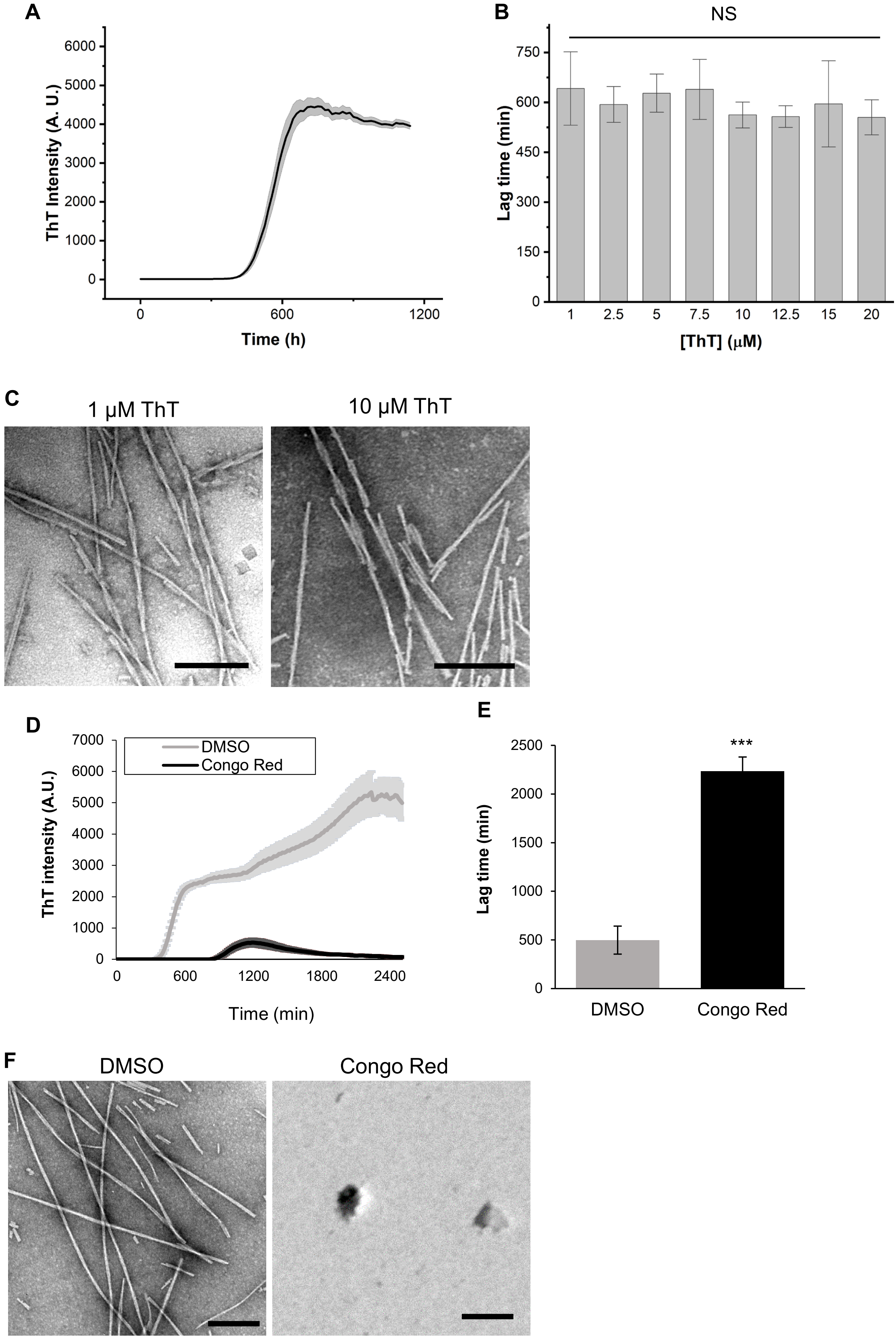

Supplementary Figure 2

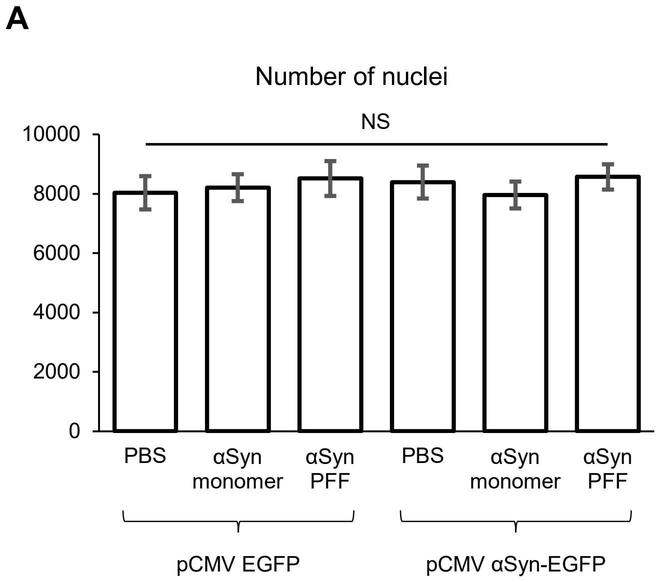

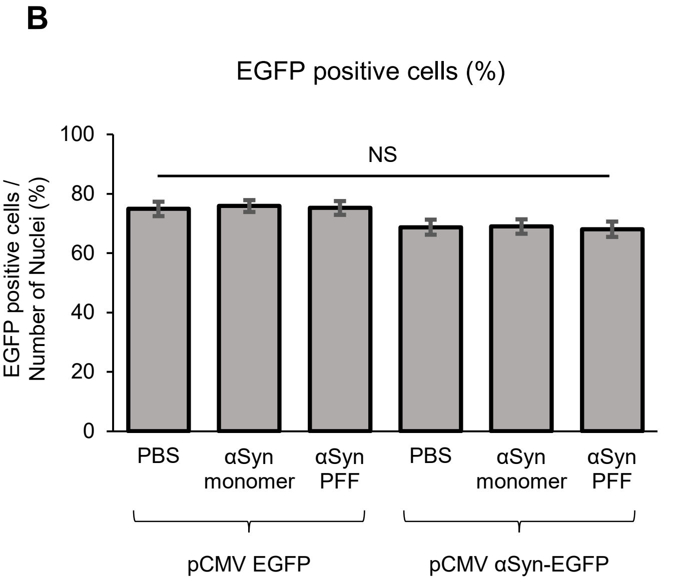

Supplementary Figure 3

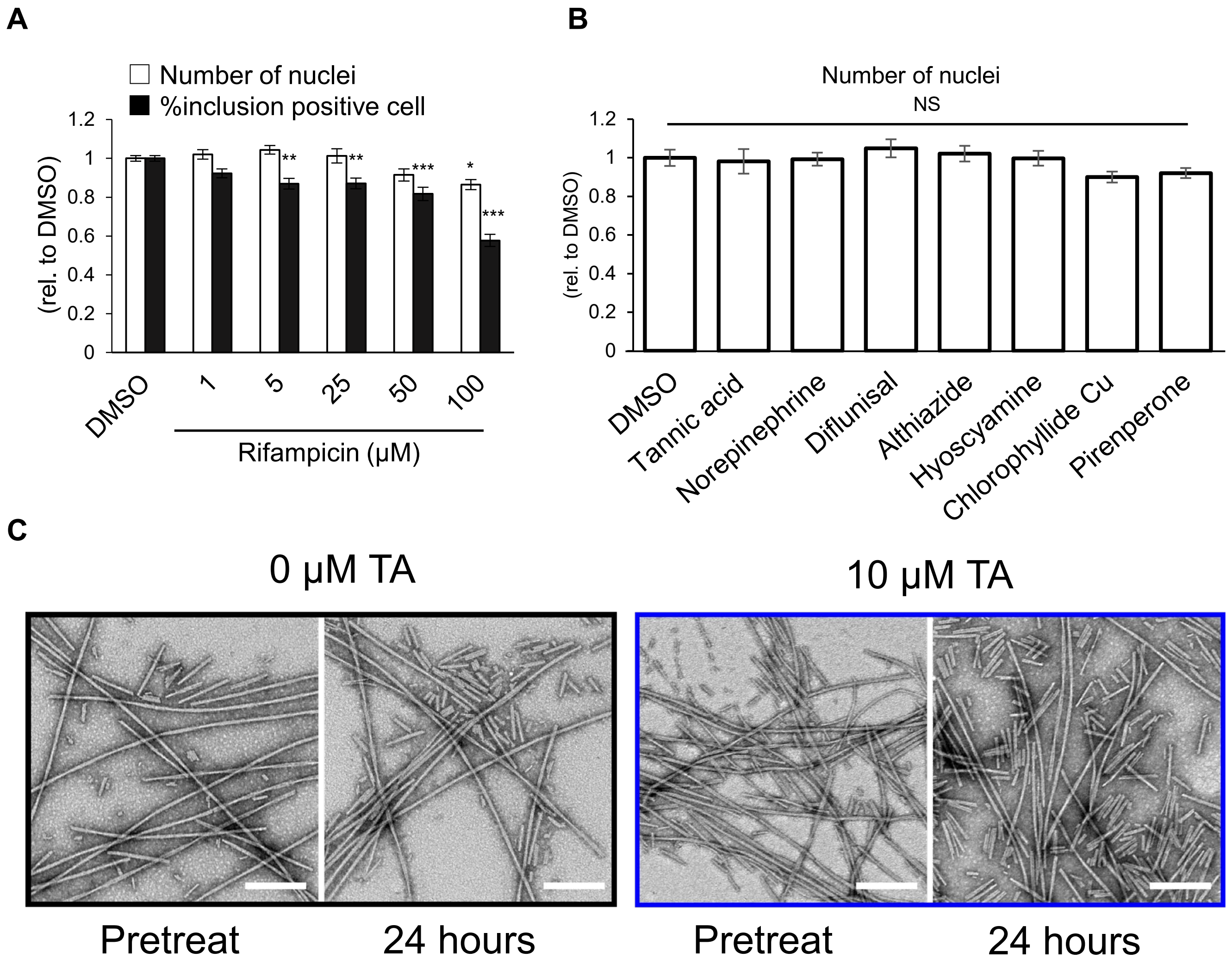

Supplement: Supplementary file 1 — Supplementary Figures. [file 41598_2021_4131_MOESM1_ESM.pdf]
